# Refining Historical Limits Method to Improve Disease Cluster Detection, New York City, New York, USA

**DOI:** 10.3201/eid2102.140098

**Published:** 2015-02

**Authors:** Alison Levin-Rector, Elisha L. Wilson, Annie D. Fine, Sharon K. Greene

**Affiliations:** New York City Department of Health and Mental Hygiene, Queens, New York, USA

**Keywords:** communicable disease, historical limits method, disease cluster detection, New York City, surveillance

## Abstract

Since the early 2000s, the Bureau of Communicable Disease of the New York City Department of Health and Mental Hygiene has analyzed reportable infectious disease data weekly by using the historical limits method to detect unusual clusters that could represent outbreaks. This method typically produced too many signals for each to be investigated with available resources while possibly failing to signal during true disease outbreaks. We made method refinements that improved the consistency of case inclusion criteria and accounted for data lags and trends and aberrations in historical data. During a 12-week period in 2013, we prospectively assessed these refinements using actual surveillance data. The refined method yielded 74 signals, a 45% decrease from what the original method would have produced. Fewer and less biased signals included a true citywide increase in legionellosis and a localized campylobacteriosis cluster subsequently linked to live-poultry markets. Future evaluations using simulated data could complement this descriptive assessment.

## Abstract 2

Our refinements corrected for major biases, preserved simplicity, and improved validity.

Detecting aberrant clusters of reportable infectious disease quickly and accurately enough for meaningful action is a central goal of public health institutions (*1*–*3*). Clinicians’ reports of suspected clusters of illness remain critical for surveillance (*4*), but the application of automated statistical techniques to detect possible outbreaks that might otherwise not be recognized has become more common (*5*). These techniques are particularly important in jurisdictions that serve large populations and receive a high volume of reports because manual review and investigation of all reports are not feasible.

Challenges such as lags in reporting and case classification and discontinuities in surveillance case definitions, reporting practices, and diagnostic methods are common across jurisdictions. These factors can impede the timely detection of disease clusters. Statistically and computationally simple methods, including historical limits (*6*), a log-linear regression model (*7*), and cumulative sums (*8*), each have strengths and weaknesses for prospective cluster detection, but none adequately address these common data challenges. As technology advances, statistically and computationally intensive methods have been developed (*2*,*3*,*5*,*9*–*12*), and although these methods might successfully correct for biases, many lack the ease of implementation and interpretation desired by health departments.

Since 1989, the US Centers for Disease Control and Prevention has applied the historical limits method (HLM) to disease counts and displayed the results in Figure 1 of the Notifiable Diseases and Mortality Tables in the Morbidity and Mortality Weekly Report (*13*). Because the method relies on a straightforward comparison of the number of reported cases in the current 4-week period with comparable historical data from the preceding 5 years, its major strengths include simplicity, interpretability, and implicit accounting for seasonal disease patterns. These strengths make it a potentially very useful aberration-detection method for health departments (*12*,*14*–*18*). The Bureau of Communicable Disease (BCD) of the New York City (NYC) Department of Health and Mental Hygiene (DOHMH) implemented the HLM in the early 2000s (HLM_original_) as a weekly analysis for all reportable diseases for which at least 5 years of historical data were available.

**Figure 1:**
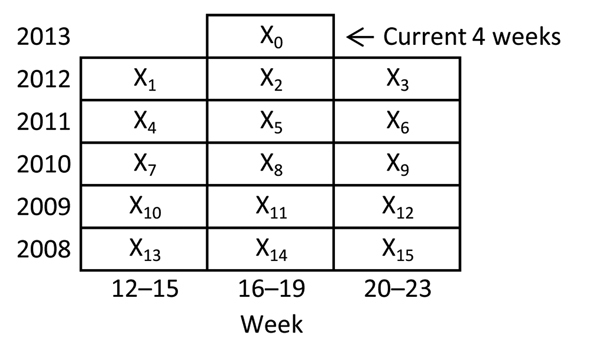
Following Stroup et al. (*21*), a schematic of the periods included in analyses using the historical limits method.

In HLM_original_, 4 major causes of bias existed: 1) inconsistent case inclusion criteria between current and historical data; 2) lack of adjustment in historical data for gradual trends; 3) lack of adjustment in historical data for disease clusters or aberrations; and 4) no consideration of reporting delays and lags in data accrual. Our objectives were to develop refinements to the HLM (HLM_refined_) that preserved the simplicity of the method’s output and improved its validity and to characterize the performance of the refined method using actual reportable disease surveillance data. Although we describe the specific process for refining BCD’s aberration-detection method, the issues presented are common across jurisdictions, and the principles and results are likely to be generalizable.

## Methods

### Overview of Disease Monitoring at BCD

BCD monitors ≈70 communicable diseases among NYC’s 8.3 million residents (*19*). For passive surveillance, laboratories and providers are required to submit disease reports (*20*), and these reports flow into a database system (Maven, Consilience Software, Austin, TX, USA). Each case is classified into 1 of 12 case statuses (Table 1). Depending on the disease, cases initially might be assigned a transient pending status and, upon investigation, be reclassified as a case (confirmed, probable, or suspected) or “not a case.” For each disease, a designated disease reviewer is responsible for reviewing cases.

**Table 1:** Case statuses in current and baseline periods included in HLM_original_ and HLM_refined_, New York City, New York, USA*

Case status	Included in HLM_original_	Included in HLM_refined_
Confirmed	Yes	Yes
Probable	Yes	Yes
Suspected	Yes	Yes
Pending†	Yes	Yes
Unresolved	No	Yes
“Not a case”	No	Yes
Chronic carrier	No	Yes
Asymptomatic infection	No	Yes
Seroconversion 1 y	No	Yes
Not applicable	No	Yes
Contact	No	No
Possible exposure	No	No

*HLM, historical limits method; HLM_original_, method as originally applied in New York City before May 20, 2013; HLM_refined_, refined method applied starting May 20, 2013. †Pending is a transient status that in the normal course of case investigations can be assigned to a case in the current period but not in the baseline period.

### HLM Overview

HLM compares the number of reported cases diagnosed in the past 4 weeks () with the number diagnosed within 15 prior periods () comprising the same 4-week period, the preceding 4-week period, and the subsequent 4-week period during the past 5 years (Figure 1). A 4-week temporal unit of analysis balances timeliness with stability (*6*,*21*). For any given disease, if the ratio of current counts to the mean of the fifteen 4-week totals is greater than historical limits, then the current period is considered aberrant (i.e., a signal is generated) (Technical Appendix). In applying this method in NYC, only increases in case counts >2 SD above the historical mean are considered because artifactual decreases in case counts would be detected by separate quality-control measures.

HLM_original_ was run each Monday for the 4-week interval that included cases diagnosed through the most recent Saturday. Data on confirmed, probable, suspected, or pending cases (Table 1) were analyzed at 3 geographic resolutions: citywide, borough (5 boroughs), and United Hospital Fund (UHF) neighborhood (42 neighborhoods). UHF neighborhoods are aggregations of contiguous ZIP codes used to define communities (*22*). Data were analyzed at the 2 subcity geographic resolutions to improve the signal-to-noise ratio for spatial clusters. For a signal to be generated, the current period was required to contain at least 3 cases, and the ratio of cases to the historical mean was required to be greater than historical limits. Disease reviewers were promptly notified of any signals and were provided with a corresponding case line list.

### Refinements to Address Biases

#### Bias 1: Inconsistent Case Inclusion Criteria

The first limitation of HLM_original_ as applied in NYC was that case inclusion criteria caused current disease counts to be systematically higher than baseline disease counts for many diseases. Cases classified as confirmed, probable, suspected, or pending were analyzed, but some cases with an initial pending status were ultimately reclassified after investigation as “not a case.” This reclassification process was complete for historical periods but ongoing for the current period.

**Figure 2:**
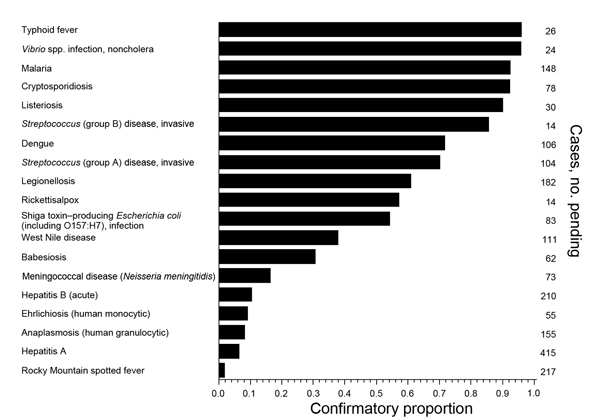
Confirmatory proportion of pending cases for diseases with any pending cases, New York City, New York, USA, July–December 2012. The confirmatory proportion was defined as the proportion of initially pending cases that were reclassified to confirmed, probable, or suspected (rather than to “not a case”). Diseases that are not routinely investigated, e.g., campylobacteriosis, enter the database with confirmed (not pending) case status and are not shown.

The proportion of initially pending cases that were reclassified to confirmed, probable, or suspected (rather than “not a case”) varied widely by disease (Figure 2). For diseases for which this confirmatory proportion was low, the disease counts in the current period included a high proportion of pending cases that would ultimately be reclassified as “not a case,” leading to false signals (type I errors). A similar bias might apply for nationally notifiable data in that provisional and final case counts may be systematically different (*23*).

#### Refinement 1: Consistent Case Inclusion Criteria

HLM_refined_ included almost all reported cases in the analysis regardless of current status (Table 1). This simple modification led to a more valid comparison of total reporting volume between current and historical periods, assuming that reporting is consistent over time, rather than biased estimates of the true level of disease. We maintained the requirement of the presence of at least 3 confirmed, probable, suspected, or pending cases to be considered a signal to prevent alerts driven by cases classified as “not a case.”

#### Bias 2: Gradual Trends in Historical Data

The second limitation of HLM_original_ was the existence of increasing or decreasing trends over time in historical data for many diseases. Whether these trends are true changes in disease incidence or artifacts of changing reporting or diagnostic practices, anything that causes disease counts in the baseline period to be systematically higher than current disease counts increases type II errors, and anything that causes baseline disease counts to be systematically lower than current disease counts increases type I errors.

#### Refinement 2: Adjusted Historical Data to Remove Gradual Trends

**Figure 3:**
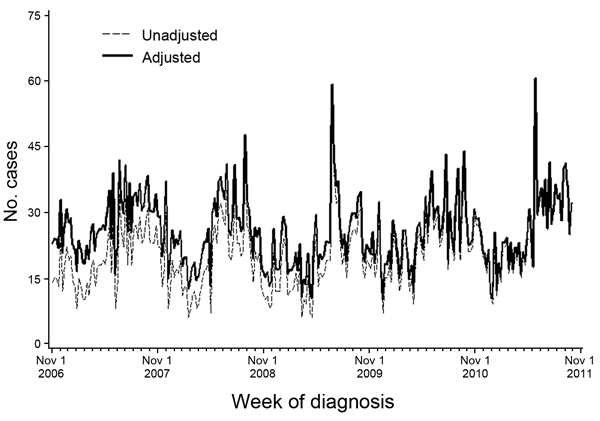
Unadjusted and adjusted weekly citywide counts of campylobacteriosis cases to illustrate adjustment for a linear trend in historical data, New York City, New York, USA, November 2006–October 2011.

For HLM_refined_, we identified and removed any significant linear trend in historical data. We accomplished this refinement by running a linear regression on weekly case counts for each disease at each geographic resolution and refitting the resulting residuals to a trend line with a slope of 0 and an intercept set to the most recent fitted value. Across diseases, linear trends were of relatively small magnitude; the greatest was for *Campylobacter*, for which the slope increased by ≈0.25 cases per week (Figure 3).

To minimize the influence of outliers on the overall trend, we excluded weekly counts >4 SD above or below the average for the baseline period from the regression. However, these counts were added back after the model had been fitted.

#### Bias 3: Inclusion of Past Clusters in Historical Data

The third major bias in HLM_original_ was the inclusion of past clusters or aberrations in historical data. This bias reduced the method’s ability to detect aberrations going forward, which increased type II errors.

#### Refinement 3: Exclusion of Past Clusters from Historical Data

**Figure 4:**
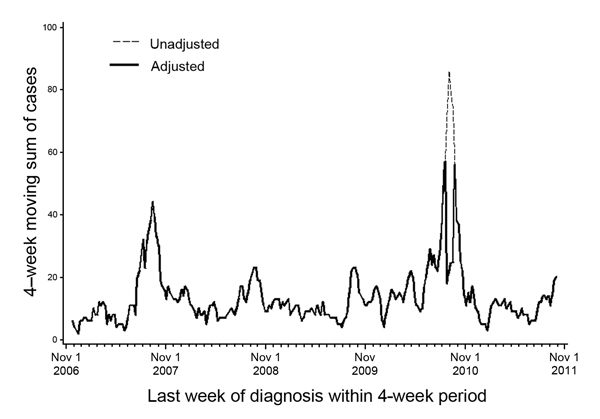
Unadjusted and adjusted 4-week moving sum of citywide dengue fever cases to illustrate adjustment for outliers in historical data, New York City, New York, USA, November 2006–October 2011.

To prevent this bias, after adjusting for gradual trends, we considered any 4-week period in which disease counts were >4 SD above the average to be an outlier and reset the count to the average number of cases in the remaining historical instances of that 4-week period. (We selected the threshold of 4 SD after manually reviewing case counts over time for all diseases.) For example, during 2007–2011, the number of dengue fever cases diagnosed during weeks 35–38 in 2010 was >4 SD above the average number of cases during those 5 years. Consequently, that 4-week period in 2010 was considered an outlier and reset to the average dengue fever count in weeks 35–38 in 2007, 2008, 2009, and 2011 (Figure 4). This technique can cause the case counts over time to appear jagged, but because our objective was to ensure a valid comparison between historical and current data, the smoothness of trends over time is irrelevant.

#### Bias 4: Delays in Data Accrual

Finally, data accrual delays can contribute to type II errors. This method is applied on Mondays for the 4-week period that includes cases diagnosed through the most recent Saturday, so any lag between diagnosis and receipt by BCD of >2 days has the potential to deflate disease counts in the current period and reduce signal sensitivity. During July 18, 2012–August 28, 2013, the median lag between diagnosis and receipt by BCD was 5 days (range in median lag by disease 0–24 days).

Although DOHMH works with laboratories and providers to improve reporting practices, substantial reporting lags will continue for some diseases because of practices related to testing (e.g., time required for culturing and identifying *Salmonella* from a clinical sample) and surveillance (e.g., for some diseases, reports are held for delivery to the surveillance database until both a positive screening test and a confirmatory test are reported).

#### Refinement 4: Repeated Analyses to Accommodate Delays in Data Accrual

For diseases for which a delay of ≥1 week is not too long for a signal to be of public health value, we repeated the analysis for a given 4-week period over 4 consecutive weeks to allow for data accrual, thus improving signal sensitivity. In other words, we first analyzed cases diagnosed during a 4-week period on the following Monday. Updated data for the same 4-week period were re-analyzed on the subsequent 4 Mondays as data accrued to identify any signals that were initially missed because of incomplete case counts.

### Customization by Disease

In HLM_original_, we conducted the same analysis for all diseases under surveillance, despite very different disease agents and epidemiologic profiles. We solicited comments from disease reviewers to ensure that the method was being applied meaningfully to all diseases and received feedback that HLM_original_ produced an unmanageable number of signals, which led to their dismissal without investigation. We also suspect that on some occasions HLM_original_ did not detect true clusters because trends in disease counts decreased over the baseline period or because historical outbreaks masked new clusters. We responded by allowing for disease-specific analytic modifications, which included reducing the number of diseases monitored using this method, allowing for customized signaling thresholds, and accounting for sudden changes in reporting (Table 2).

**Table 2:** Diseases included in analyses using HLM_refined_ and details of customizations, New York City, New York, USA, May 20–August 5, 2013*

Disease	Minimum no. cases in UHF neighborhood to qualify for signal	Further customization
Amebiasis	5	
Anaplasmosis (human granulocytic)	3	
Babesiosis	3	
Campylobacteriosis	8	
Cholera	3	
Cryptosporidiosis	5	
Cyclosporiasis	3	
Dengue	3	
Ehrlichiosis (human monocytic)	3	
Giardiasis	5	
*Haemophilus influenzae* disease, invasive	3	
Hemolytic uremic syndrome	3	
Hepatitis A	5	
Hepatitis B (acute)	2†	
Hepatitis D	2†	
Hepatitis E	2†	
Legionellosis	5	
Listeriosis	3	
Malaria	3	
Meningitis, bacterial	4	
Meningitis, viral (aseptic)	3	
Meningococcal disease (*Neisseria meningitidis*)	3	
Paratyphoid fever	3	
Rickettisalpox	3	
Rocky Mountain spotted fever	3	Restrict analysis to confirmed, probable, and suspected cases and implement a 4-wk lag to allow for data accrual
Shiga toxin–producing *Escherichia coli* (including *E. coli* O157:H7) infection	3	
Shigellosis	10	
*Staphylococcus aureus* infection, vancomycin intermediate	3	
*Streptococcus* (group A) disease, invasive	5	Restrict analysis to confirmed, probable, suspected, and pending cases
*Streptococcus* (group B) disease, invasive	5	
*Streptococcus pneumoniae* disease, invasive	5	
Typhoid fever	3	
*Vibrio* spp. infection, noncholera (including *parahaemolyticus* and *vulnificus*)	3	
West Nile disease	3	
Yersiniosis	3	

* HLM_refined_, refined method applied starting May 20, 2013; UHF, United Hospital Fund. †These are the only diseases for which the signaling threshold was decreased below 3 cases.

We reduced the ≈70 diseases to which HLM_original_ had been applied to the 35 for which prospective and timely identification of clusters might result in public health action. For example, clusters of leprosy or Creutzfeldt-Jakob disease diagnoses within a 4-week period would not be informative because these diseases have long incubation periods, measured in years. We also excluded diseases that occur very infrequently or are nonexistent (defined as having an annual mean of <4 cases during 2008–2012). For example, we excluded tularemia and human rabies because any clusters of these diseases would be detected without automated analyses and because the underlying normality assumption of the method is violated for rare events.

Signals were most common at the neighborhood geographic level because of the increased noise resulting from small counts. Therefore, we also provided the option to reviewers to require >3 confirmed, probable, suspected, or pending cases to qualify as a signal at this geographic resolution.

### Evaluation of HLM_refined_

BCD implemented HLM_refined_ on May 20, 2013, including automatically generating reports for disease reviewers to summarize information about cases included in signals (online Technical Appendix). To determine the effects of the above refinements, we compared signals detected during the 12 weeks after implementation with those that would have been detected had HLM_original_ still been in place. A signal was defined as any set of consecutive 4-week periods, permitting 1-week gaps, where the disease counts were above historical limits for either HLM_original_ or HLM_refined_. Signals that were repeated in the same geographic area over multiple consecutive weeks were counted only once. Restricting analysis to a common set of 35 diseases (Table 2), we quantified the number of signals, determined the cause of any differences in signals between HLM_original_ and HLM_refined_, and monitored the outcome of any public health investigations triggered by automated signals.

We describe our experience with these methods in a government setting to support applied public health practice. In this setting, a complete list of true disease clusters and the resources to thoroughly investigate every statistical signal do not exist. We instead defined the set of true disease clusters as those identified using either method that could not be explained by any known systematic bias. We calculated type I and type II error rates using this set. Although artificial surveillance data generated through simulations have been created (*24*,*25*), those existing data do not reflect the dynamism and variability in actual reportable disease surveillance data, such as pending case reclassification (bias 1) and data accrual lags (bias 4). Accounting for this dynamism is essential for a valid comparison of HLM_original_ and HLM_refined_. Thus, we chose a practical and descriptive approach to evaluating these methods rather than a quantitative simulation study.

## Results

In the first 12 weekly analyses, HLM_original_ would have produced 134 signals, and HLM_refined_ produced 74 signals, a 45% decrease (Table 3). Of the HLM_original_ signals during this period, 47 (35%) would have been at the neighborhood geographic resolution with fewer cases than the reviewers’ threshold for action; these signals were omitted from further evaluation. Of the remaining 107 signals across both methods, 54 (50%) were detected by both methods, 33 (31%) only by HLM_original_, and 20 (19%) only by HLM_refined_.

**Table 3:** Geographic resolution of signals produced by HLM_original_ and HLM_refined_ in 12 weekly analyses, New York City, New York, USA, May 20–August 5, 2013*

Geographic area	≥3 Cases required for signal: No. signals produced by HLM_original_	No. signals produced by HLM_original_†	No. signals produced by HLM_refined_†
City	14	14	8
Borough	40	40	26
UHF neighborhood	80	33	40
Total	134	87	74

*HLM, historical limits method; HLM_original_, method as originally applied in NYC prior to May 20, 2013; HLM_refined_, refined method applied starting May 20, 2013; UHF, United Hospital Fund. †At neighborhood level, reviewer could require >3 cases for signal.

We classified each signal into 1 of 3 categories (Table 4): attributable to an uncorrected bias toward signaling, attributable to the correction of a bias against signaling, or not attributable to any known systematic bias. Of the signals detected by HLM_original_, 2 campylobacteriosis signals and 1 invasive *Haemophilus influenzae* disease signal were attributable to a bias toward signaling caused by an increasing trend in historical data. HLM_refined_ missed 9 signals that were detected only by HLM_original_ because the confirmatory proportion was larger in current data than in historical data.

**Table 4:** Explanation of signals produced by HLM_original_ and HLM_refined_ in the 12 weekly analyses, New York City, New York, USA, May 20–August 5, 2013*

Explanation	No. signals produced by HLM_original_	No. signals produced by HLM_refined_
Attributable to an uncorrected bias toward signaling		
Neighborhood disease count threshold too low	47†	0
Pending cases in current period	21	0
Increasing trends in baseline period	3	0
Total signals attributable to an uncorrected bias toward signaling	71	0
Attributable to the correction of a bias against signaling		
Confirmatory proportion higher in current period than in baseline period	9	0
Accounted for data accrual lags	0	17
Deleted outliers in baseline period	0	2
Adjusted for decreasing trends in baseline period	0	1
Total signals attributable to the correction of a bias against signaling	9	20
Not attributable to any known systematic bias	54	54
Total signals	134	74

*HLM, historical limits method; HLM_original_, method as originally applied in NYC prior to May 20, 2013; HLM_refined_, refined method applied starting May 20, 2013. †These were excluded from the calculation of type I and type II error rates.

Two signals detected by HLM_refined_ were attributable to the removal of outliers from historical data; a legionellosis increase in the Bronx was masked by a prior increase in comparable weeks in 2009, and an amebiasis signal in a neighborhood was masked by a prior increase in comparable weeks in 2012. One signal detected by HLM_refined_ was attributable to the adjustment of a decreasing trend in baseline disease counts of viral meningitis. Seventeen signals detected only by HLM_refined_ were attributable to accounting for lags in data accrual (10 signals were first detectable after 1-week lag, 4 signals after 2 weeks, 2 signals after 3 weeks, and 1 signal after 4 weeks).

Overall, we identified 83 true clusters that could not be explained by any known systematic bias (i.e., 54 clusters identified by both HLM_original_ and HLM_refined_ and 29 clusters detected by only 1 of the methods and attributable to the correction of a bias against signaling). During the evaluation period, the percentage of all signals that did not correspond to these true clusters (type I error rate) for HLM_original_ was 28% (24 of 87 signals) and, for HLM_refined_, 0% (0 of 74 signals). The percentage of all true clusters that were not detected (type II error rate) for HLM_original_ was 24% (20 of 83 true clusters) and, for HLM_refined_, 11% (9 of 83 true clusters).

During these 12 weeks, 2 disease clusters occurred that we would have expected to detect using HLM. The first cluster of interest was a citywide increase in legionellosis in June 2013 (*26*). HLM_refined_ first detected this increase with a cluster in Queens on June 24, 2013. The next week, both HLM_refined_ and HLM_original_ detected the citywide increase. Although HLM_refined_ and HLM_original_ might detect similar disease clusters at slightly different times because of differences in event inclusion criteria, the refinements do not directly affect timeliness.

On June 24, 2013, HLM_original_ would have generated 16 automated signals (including 3 for campylobacteriosis), and HLM_refined_ generated 5 signals (including 1 for campylobacteriosis); both methods detected a cluster of 11 campylobacteriosis cases in 1 neighborhood. After investigation, 8 of the cases were determined to be among children 0–5 years of age from Mandarin- or Cantonese-speaking families, 5 of whom had direct links to 1 of 2 local live-poultry markets. Consequently, pediatricians were educated about the association between live-poultry markets and campylobacteriosis, and health education materials about proper poultry preparation and hygiene were distributed to live-poultry markets.

## Discussion

In refining the HLM to correct for major biases, we improved the ability to prospectively detect clusters of reportable infectious disease in NYC while preserving the simplicity of the output. Specifically, we addressed data challenges that are common to many jurisdictions, including improving consistency of case inclusion criteria, accounting for gradual trends and aberrations in historical data, and accounting for reporting delays.

HLM_refined_ found fewer signals overall than HLM_original_, which, in practice, is perhaps the greatest improvement. Disease reviewers had become accustomed to a large number of signals that did not represent true outbreaks, which led to dismissal of many signals without investigation. Fewer, higher quality signals produced by HLM_refined_, supported by improvements in the ad hoc type I and type II error rates, led to more careful inspection and a higher probability of identifying true clusters, e.g., the true campylobacteriosis cluster in a Brooklyn neighborhood.

Although we consider HLM_refined_ to be a substantial improvement upon HLM_original_, we are aware that some limitations exist. In expanding case inclusion criteria to encompass all reports, we corrected a large bias but might have introduced a small bias. Because HLM_refined_ considers the overall volume of reported cases, the implicit assumption is that the confirmatory proportion is constant over time outside of seasonal patterns. If this assumption is violated, and the confirmatory proportion differs between historical and current data, HLM_refined_ can be biased. This bias is the reason that 9 signals detected by HLM_original_ were not also detected by HLM_refined_ during the evaluation period. Because these 9 signals might reflect disease clusters that would have been missed because of changes in the confirmatory proportion over time, we recommend implementing a lagged analysis that is restricted to confirmed, probable, and suspected cases. The signals produced by this lagged analysis can then be compared with signals produced in near real-time using all case statuses, and thus whether HLM_refined_ systematically fails to detect clusters can be assessed. Implementing this approach post hoc yielded 2 additional clusters that both HLM_refined_ and HLM_original_ missed. Also, as with any method that defines geographic location according to patient residence, HLM_refined_ can miss point source outbreaks when exposure occurs outside the residential area.

Next steps include addressing the arbitrary temporal and geographic units of analysis. HLM_refined_ is optimized to detect clusters of 4-week duration at citywide, borough, or neighborhood geographic resolution. This method is likely to fail to detect clusters of shorter or longer duration, at sub-neighborhood geographic resolution, and in locations that span borough or neighborhood borders. In February 2014, we began applying the prospective space–time permutation scan statistic so we could use flexible spatial and temporal windows (*27*). We plan to expand the application of HLM_refined_ to disease subspecies and serogroups within diseases (e.g., for salmonellosis) as this information becomes available in BCD’s database system.

Health departments that receive a high volume of reports might consider adopting a method similar to HLM_refined_ to improve prospective outbreak detection and contribute to timely health interventions. Simulation studies using complex artificial data that adequately reflect the dynamic nature of real-time surveillance data across a wide range of reportable diseases with variable trends over time and historical outbreaks would be valuable.

Technical Appendix Supplemental information, SAS code, and sample output for the refined historical limits method, New York City, New York, USA.
