# Supplementary material for: Refining Historical Limits Method to Improve Disease Cluster Detection, New York City, New York, USA
Source: Emerg Infect Dis. 2015 Feb;21(2):265–72. doi: 10.3201/eid2102.140098 (PMC4313630; doi:10.3201/eid2102.140098)
Supplement: Technical Appendix — Supplemental information, SAS code, and sample output for the refined historical limits method, New York City, New York, USA. [file 14-0098-Techapp-s1.pdf]

Article DOI: http://dx.doi.org/10.3201/eid2102.140098

Refining Historical Limits Method to Improve Disease Cluster Detection, New York City, New York, USA

Technical Appendix

The Technical Appendix contains mathematical notation for the HLM, details on customized analyses implemented for 2 diseases with unique patterns of reporting and diagnosis over time, a technical note, sample output for summarizing and presenting signals, and sample SAS code (SAS v.9.2, SAS Institute, Cary, NC).

Mathematical Notation for HLM

$$\frac{X_0}{\mu} > 1 + 2 * \left(\frac{\sigma_x}{\mu}\right) \quad (1)$$

where X_0 is the current total of cases in the most recent four-week interval, and μ and σ_x are the mean and standard deviation, respectively, of the 15 historical four-week periods (X_{1-15}).

Customized Analyses for Two Diseases

For Group A *Streptococcus*, new filtering rules in our surveillance database system were applied in July 2012 to screen out reports of specimens collected from noninvasive sources, resulting in an abrupt decrease in the number of reported cases. Ignoring this change would have biased against signaling. Therefore, we continued to consider only cases with confirmed, probable, suspected, or pending statuses. Because the confirmatory proportion is high for this disease (73%, Figure 2), including pending cases does not strongly bias toward signaling.

For Rocky Mountain Spotted Fever, the total number of reported cases increased beginning in summer 2011, while there was no corresponding increase in the number of confirmed, probable, and suspected cases. Ignoring this pattern would have biased toward false signaling. We therefore monitor only confirmed, probable, and suspected cases at a lag of four

weeks to allow for near complete data accrual. Signal quality was prioritized over timeliness for this disease, which has no immediate public health intervention.

Technical Note

Since these adjustments of the baseline data require a time series in which to identify outliers, calculate averages, and run regressions, baselines will need to be prospectively updated at regular intervals. It is not necessary to recalibrate historical data on a weekly basis because the adjustments will not be very different from week to week. However, the interval at which recalibrations are made should be sufficiently short such that recent disease trends are taken into account. The interval must be shorter than one year because data less than a year in the past is considered as the most recent historical data point in the method (X_3 in Figure 1), and we want at least 12 weeks to have passed between the end of the baseline period and the date of recalibration to allow for sufficient data accrual. We chose this 12 week cutoff based on the fact that in 2010 and 2011, all relevant diseases had at least 70% data accrual at 12 weeks post diagnosis, and all but two diseases (encephalitis and human granulocytic anaplasmosis) had at least 90% data accrual.

It is these considerations that led us to conclude that the baseline data should be recalibrated every 26 weeks (twice per year), e.g., on the 1st and 27th weeks of the year, and include historical data from the earliest week that is required for comparison by the method (X_{13} in Figure 1) through 12 weeks prior to the recalibration date. In other words, in the baseline period to be used for prospective surveillance during weeks 1 through 26 of year *Y*, the earliest week that is required is week 46 of year *Y*-6 (the 4-week period from week 46 through week 49 of year *Y*-6 constitutes time period X_{13} for week 1 of year *Y*). The latest week that is included is week 40 of year *Y*-1 (12 weeks prior to week 1 of year *Y*). Analogously, the baseline period for weeks 27 through 52 of year *Y* will include week 20 of year *Y* -5 through week 14 of year *Y*.

Sample SAS Code for Implementing Refinements 2 and 3

The following sample SAS code adjusts historical data to remove gradual trends and resets outliers that could indicate past clusters to the average number of cases in the remaining instances of that 4-week period in historical data.

Structure your historical dataset called collapsed_events_city in the following format. The variable “fsatdiag” refers to the Saturday ending the week of interest (i.e. 5/17/2008 refers to the week from Sunday, 5/11/2008, through Saturday, 5/17/2008, inclusive). Ensure that every Saturday is included in the dataset for each disease and geographic area, even if the number of events for that week is zero. If you are running an analysis at a smaller geographic area, include another variable to identify the disease count within each area.

Sample structure for input dataset, named “collapsed_events_city”

Disease_code	Disease	Events	fsatdiag
Dis1	Disease1	15	5/17/2008
Dis1	Disease1	4	5/24/2008
…	…	…	…
Dis2	Disease2	0	5/17/2008

```
********************************************************************;
*     PROGRAM NAME: Adjusting Baseline for HLM Refined
*       PROGRAMMER: Alison Levin-Rector
********************************************************************;

*-- assign libnames;
libname signals 'SPECIFY LOCATION TO SAVE ADJUSTED BASELINE DATA';

* 1. REMOVE GRADUAL TRENDS FROM HISTORICAL DATA;
* weekly event count is excluded from the regression model if it is more than
4 standard deviations greater or less than the mean of entire dataset, to
avoid biasing trend;
*-- define the baseline period depending on whether we are in the first or
second half of the calendar year;
data _null_;
      if week(date()) <= 26 then firstday = nwkdom(1,7,1,year(date())-
            6)+45*7;
      if week(date()) <= 26 then lastday = nwkdom(1,7,1,year(date())-1)+39*7;
      if week(date()) > 26 then firstday = nwkdom(1,7,1,year(date())-5)+19*7;
      if week(date()) > 26 then lastday = nwkdom(1,7,1,year(date()))+13*7;
      call symputx ('firstday_BL',firstday);
      call symputx ('lastday_BL',lastday);
run;

*-- exclude weeks with event counts that are more than 4 standard deviations
above or below the mean for that disease from the regression;
proc sql;
      create table collapsed_events_outliers as
select *, mean(events) + 4*std(events) as cutoff1, mean(events) -
      4*std(events) as cutoff2
      from collapsed_events_city
where fsatdiag <= &lastday_BL
      group by disease_code
      order by disease_code, fsatdiag;
quit;
```

```
data collapsed_events_outliers;
      set collapsed_events_outliers;
      if events <= cutoff1 & events >= cutoff2 then events_model = events;
run;
*-- run regression;
proc reg data = collapsed_events_outliers;
      by disease_code;
      model events_model = fsatdiag;
      output out = reg_output r = res p = pred;
      ods output ParameterEstimates = params;
run;
*-- save the number of events predicted by the linear regression at the most
recent week for each disease;
proc sql;
      create table newline as
      select disease_code, pred as new_intercept
      from reg_output
      having max(fsatdiag) = fsatdiag
      order by disease_code;
quit;
*-- save the p-values from slope term to determine whether the regression
found a significant trend for each disease;
proc sql;
      create table slopes as
      select disease_code, probt
      from params
      where strip(variable) = "fsatdiag"
      order by disease_code;
quit;
*-- if the trend had a significant slope, then adjust event counts so that
the trend over time is flat and if not, preserve the original event counts;
data adjusted_events_sig;
      merge reg_output slopes newline;
      by disease_code;
      res2 = events-pred;
      events_adj = new_intercept + res2;
      if probt > .05 | probt = . then events_adj = events;
run;

* 2. EXCLUDE PAST CLUSTERS FROM HISTORICAL DATA;
* This step is applied to 4-week periods rather than weekly counts, so first
we create a moving 4-week sum of events over our entire baseline period;
data adjusted_events_sig_outliers;
      set adjusted_events_sig;
      by disease_code;
      retain num_sum num_sum_adj 0;
      if first.disease_code then do;
      count=0;
            num_sum=0;
            num_sum_adj=0;
      end;
      count+1;
      last4=lag4(events);
      if count gt 4 then num_sum=sum(num_sum,events,-last4);
      else num_sum=sum(num_sum,events);
      last4adj=lag4(events_adj);
      if count gt 4 then num_sum_adj=sum(num_sum_adj,events_adj,-last4adj);
```

```
      else num_sum_adj=sum(num_sum_adj,events_adj);
      drop last4 last4adj;
run;
proc sql;
      create table adjusted_events_sig_outliers as
      select disease_code, disease, fsatdiag, num_sum, num_sum_adj,
            significant, mean(num_sum_adj) + 4*std(num_sum_adj) as cutoff
      from adjusted_events_sig_outliers
      where count >= 4
      group by disease_code
      order by disease_code, fsatdiag;
quit;
*-- require more than 2 events to be considered an outlier;
data adjusted_events_sig_outliers;
      set adjusted_events_sig_outliers;
      num_sum_outliers = num_sum_adj;
      if num_sum_adj >= cutoff & num_sum_adj > 2 then num_sum_adj = .;
      week = week(fsatdiag) + 1;
run;
*-- fill in dropped outliers with average of the same week from other years;
proc sql;
      create table averages as
      select disease_code, week, avg(num_sum_adj) as num_sum_adj_avg
      from adjusted_events_sig_outliers
      group by disease_code, week
      order by disease_code, week;
quit;
proc sort data = adjusted_events_sig_outliers; by disease_code week; run;
data adjusted_events_sig_fill;
      merge adjusted_events_sig_outliers averages;
      by disease_code week;
      if num_sum_adj = . then num_sum_adj = num_sum_adj_avg; /* this is where
            we fill them in*/
      drop week num_sum_adj_avg;
run;
*-- save the adjusted historical data to memory;
proc sort data = adjusted_events_sig_fill out =
signals.adjusted_baseline_city;
by disease_code fsatdiag;
run;
```

Sample Output for Summarizing and Presenting Signals

The following sample output is an example of the presentation of a signal for one disease and geographic resolution. SAS code used to produce this output is provided in the subsequent section. This output is automatically generated and placed in a secured folder. The location of this output is then sent by e-mail to the appropriate disease reviewer for each signal. Not

included in this sample output is a summary of all signals produced each week that is distributed to the entire Bureau of Communicable Disease.

Campylobacteriosis

UHF Signal in Neighborhood X

Disease	Unit of geography	Date of interest	Total dx past 4 weeks: 26MAY13 - 22JUN13	Signal Strength (# of SDs above mean)	new signal since last week?	if not new, how many new events in signal?	Rate per 100,000 in signal area past 4 weeks	Citywide rate per 100,000 past 4 weeks
Campylobacteriosis	Neighborhood X	Diagnosis date	11	5.70	yes	N/A	8.6	1.55

Total dx past 4 weeks includes only confirmed, probable, suspect and pending case statuses
When # of SDs above mean > 2, current period is considered a signal
A missing signal strength value indicates an SD = 0 in the baseline period

Year	Most recent week (Sun-Sat)	2 weeks ago	3 weeks ago	4 weeks ago	Total
2013 Pending	0	0	0	0	0
2013 Conf/Prob/Susp	4	2	3	2	11
2012 Conf/Prob/Susp	3	1	1	0	5
2011 Conf/Prob/Susp	1	1	1	0	3
2010 Conf/Prob/Susp	0	0	3	0	3
2009 Conf/Prob/Susp	1	0	0	1	2
2008 Conf/Prob/Susp	0	1	1	0	2

Unadjusted counts of cases by year, week and case status

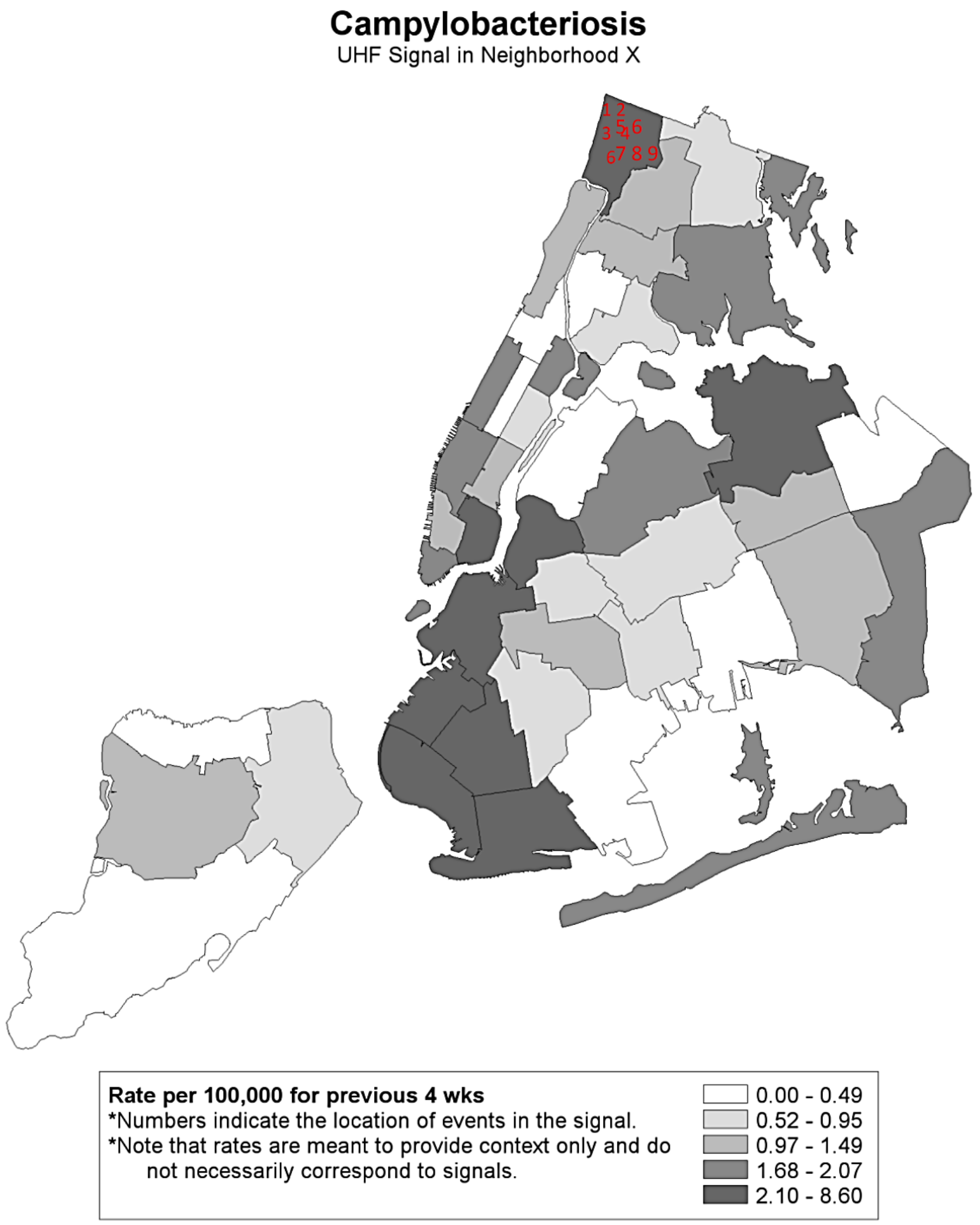

Technical Appendix Figure 1. Spatial distribution of the address at time of report for cases included in the signal and rates of disease by UHF neighborhood in the previous 4 weeks.

Case locations have been moved to a different neighborhood to protect confidentiality.

Publisher: CDC; Journal: Emerging Infectious Diseases
Article Type: Research; Volume: 21; Issue: 2; Year: 2015; Article ID: 14-0098
DOI: 10.3201/eid2102.140098; TOC Head: Dispatch

#	Event ID	Pat. init.	Disease status	Investigation status	Diagnosis date	Gender	Age	Address	Boro	Zip	UHF	Geocode
1	XXXXXX	XX	CONFIRMED	NOT_NEEDED	05/28/2013	XXXXX	N/A	XXXXXX	XX	XXXXX	Neighborhood X	yes
2	XXXXXX	XX	CONFIRMED	NOT_NEEDED	05/29/2013	XXXXX	N/A	XXXXXX	XX	XXXXX	Neighborhood X	yes
3	XXXXXX	XX	CONFIRMED	NOT_NEEDED	06/04/2013	XXXXX	N/A	XXXXXX	XX	XXXXX	Neighborhood X	yes
4	XXXXXX	XX	CONFIRMED	NOT_NEEDED	06/05/2013	XXXXX	N/A	XXXXXX	XX	XXXXX	Neighborhood X	yes
5	XXXXXX	XX	CONFIRMED	NOT_NEEDED	06/06/2013	XXXXX	N/A	XXXXXX	XX	XXXXX	Neighborhood X	yes
6	XXXXXX	XX	CONFIRMED	NOT_NEEDED	06/12/2013	XXXXX	N/A	XXXXXX	XX	XXXXX	Neighborhood X	yes
7	XXXXXX	XX	CONFIRMED	NOT_NEEDED	06/13/2013	XXXXX	N/A	XXXXXX	XX	XXXXX	Neighborhood X	yes
8	XXXXXX	XX	CONFIRMED	NOT_NEEDED	06/17/2013	XXXXX	N/A	XXXXXX	XX	XXXXX	Neighborhood X	yes
9	XXXXXX	XX	CONFIRMED	NOT_NEEDED	06/17/2013	XXXXX	N/A	XXXXXX	XX	XXXXX	Neighborhood X	yes
10	XXXXXX	XX	CONFIRMED	NOT_NEEDED	06/18/2013	XXXXX	N/A	XXXXXX	XX	XXXXX	Neighborhood X	no
11	XXXXXX	XX	CONFIRMED	NOT_NEEDED	06/21/2013	XXXXX	N/A	XXXXXX	XX	XXXXX	Neighborhood X	no

Identifying information is suppressed to protect confidentiality.

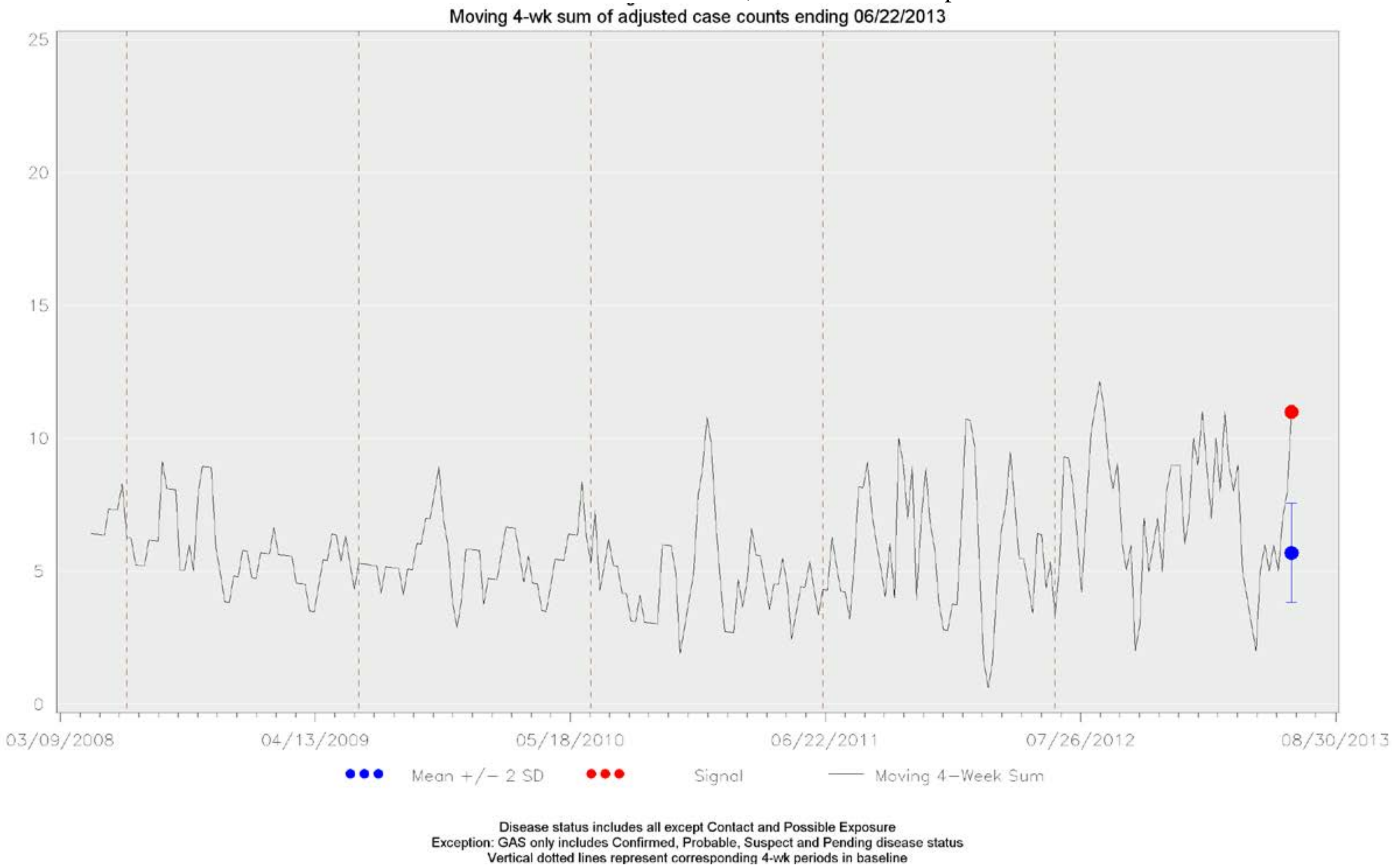

Technical Appendix Figure 2. Moving 4-week sum of adjusted case counts compared with historical mean ± 2 SD.

Sample SAS Code for Summarizing and Presenting Signals

The following sample SAS code applies the HLM method and creates an output document with a summary of all signals as well as a detailed linelist report for each signal detected (see sample output above). Also included is code that automates the emailing of signal details to reviewers.

The input dataset should be event-level data in the following structure. The variable "fsatdiag" refers to the Saturday following the diagnosis date for each event. The following code is only for a citywide analysis. If you are running an analysis at multiple geographic resolutions, then include another variable to indicate the geographic unit. The "confirmatory" variable is an indicator variable for disease status that is set to 1 if the case is confirmed, probable, suspected, or pending and 0 if it is not. The input dataset should also include variables such as patient initials, disease status, diagnosis date, gender, and age for display in linelists, and X and Y coordinates for mapping.

Sample structure for input dataset, named "event_level_input"

Disease_code	Disease	Event_ID	fsatdiag	confirmatory
Dis1	Disease1	XXXXX1	5/17/2008	1
Dis1	Disease1	XXXXX2	5/24/2008	0
…	…	…	…	…
Dis2	Disease2	XXXXX4	5/17/2008	1

```
*********************************************************************;
*     PROGRAM NAME: Analysis and Output for HLM Refined
*      PROGRAMMERS: Alison Levin-Rector
*                   Elisha Wilson
*                   Deborah Kapell
*********************************************************************;

* create macro variables for the most recent Saturday in the dataset and
today's date;
proc sql noprint;
      select max(fsatDiag)
      into :maxFSATDiag
      from event_level_input;
quit;

data _null_;
call symput ('fileweek',put(today(),date9.));
run;
```

```
***** CITYWIDE *****;
* pull in recent data (since the end of the baseline period);
proc sql;
      create table current_data as
      select disease_code, disease, fsatdiag, count(event_id) as events_adj,
            count(confirmatory) as confirmatory
      from event_level_input
      where fsatdiag > (&lastday_BL - 28)
      group by disease_code, disease, fsatdiag
      order by disease_code, disease, fsatdiag;
quit;
* merge current data with baseline data and categorize weeks into relevant
time periods for analysis;
data trends1;
      set signals.adjusted_baseline_city (rename=(num_sum_adj = events_adj))
            current_data;
      if fsatdiag >= (&maxfsatdiag-22) & fsatdiag <= &maxfsatdiag then
            period='current';
      if abs(fsatdiag - (&maxfsatdiag-365-28)) <= 3 then period='p1';
      if abs(fsatdiag - (&maxfsatdiag-365)) <= 3 then period='c1';
      if abs(fsatdiag - (&maxfsatdiag-365+28)) <= 3 then period='s1';
      if abs(fsatdiag - (&maxfsatdiag-365*2-28)) <= 3 then period='p2';
      if abs(fsatdiag - (&maxfsatdiag-365*2)) <= 3 then period='c2';
      if abs(fsatdiag - (&maxfsatdiag-365*2+28)) <= 3 then period='s2';
      if abs(fsatdiag - (&maxfsatdiag-365*3-28)) <= 3 then period='p3';
      if abs(fsatdiag - (&maxfsatdiag-365*3)) <= 3 then period='c3';
      if abs(fsatdiag - (&maxfsatdiag-365*3+28)) <= 3 then period='s3';
      if abs(fsatdiag - (&maxfsatdiag-365*4-28)) <= 3 then period='p4';
      if abs(fsatdiag - (&maxfsatdiag-365*4)) <= 3 then period='c4';
      if abs(fsatdiag - (&maxfsatdiag-365*4+28)) <= 3 then period='s4';
      if abs(fsatdiag - (&maxfsatdiag-365*5-28)) <= 3 then period='p5';
      if abs(fsatdiag - (&maxfsatdiag-365*5)) <= 3 then period='c5';
      if abs(fsatdiag - (&maxfsatdiag-365*5+28)) <= 3 then period='s5';
run;
*-- carry out HLM analysis at citywide level;
proc sql;
      create table City1 as
      select disease_code, disease, period, sum(events_adj) as count
      from trends1
      where period ^=''
      group by disease_code, disease, period;
quit;
* count the number of confirmed/probable/suspect/pending cases;
proc sql;
      create table confirmatory1 as
      select disease_code, disease, sum(confirmatory) as confirmatory
      from trends1
      where period = "current"
      group by disease_code, disease, period;
quit;
proc transpose data=City1 out=City2;
      by disease_code disease;
      id period;
      var count;
run;
data City2;
```

```
      merge City2 confirmatory1;
      by disease_code disease;
run;
data City3;
      set City2;
      array xx  current p1 c1 s1 p2 c2 s2 p3 c3 s3 p4 c4 s4 p5 c5 s5;
            do over xx;
                  if xx=. then xx=0;
            end;
      if current>0 then do;
            mean=mean(p1,c1,s1,p2,c2,s2,p3,c3,s3,p4,c4,s4,p5,c5,s5);
            sd=   std(p1,c1,s1,p2,c2,s2,p3,c3,s3,p4,c4,s4,p5,c5,s5);
            if sd>0 then ratio= (current-mean)/sd;
            if current >=mean+2*(sd) then significant=1;
            else significant = 0;
      end;
      format mean 5.1;
      format sd 5.2;
      format ratio 5.2;
      length geography $20.;
      geography= 'City';
      geoUnit='City';
      metric='Diagnosis date';
run;

** The equivalent analysis above is carried out at all geographic resolutions
(in our case at Borough and UHF neighborhood);

* merge significant signals at all geographic resolutions;
data AllSignificant;
      set City3 Boro3 UHF3;
      /* only keep signals that are significant and that have at least 3
      confirmed, probable, suspected, or pending events */
      if (confirmatory>2 & significant=1);
      fsatDiag=&maxfsatDiag;
      rundate=today();
      format fsatDiag rundate mmddyy10.;
run;
proc sort data=allsignificant;
      by disease_code geography;
run;

* delete saved signals if the analysis is run multiple times on the same day;
data signals.trends_signals;
      set signals.trends_signals;
      where rundate ~= date();
run;
* create macro variable with the last time the analysis was run;
proc sql noprint;
      select max(rundate,date9.)
      into :lastweek
      from signals.trends_signals;
quit;

* save signal details datasets;
proc append base=signals.trends_signals data=allsignificant;
```

```
run;

* compare this week's signals with last week's signals in order to flag new
signals;
proc sort data = signals.trends_signals out = lastweek (keep=disease_code
geography);
      where rundate = input("&lastweek",date9.);
      by disease_code geography;
run;
data Allsignificant_compare;
      merge allsignificant (in=a) lastweek (in=b);
      by disease_code geography;
      if a;
      if ~b then new = "*";
run;

*-- Output the signal summary document;
data health2;
confidential="Please do not Distribute";
run;
ods noresults;
ods rtf file= "…\&fileweek\Weekly trends &fileweek..doc";
title1 'TO:  STAFF';
title2 'SUBJECT:    WEEKLY TRENDS';
title3 '
';
title4 'As always, comments and feedback much appreciated.';
title5 '
';
title6 'Thanks !';
proc print data=health2 noobs;
var confidential;
run;

options orientation=landscape;
proc print data=allsignificant_compare noobs label  ;
var disease  geography confirmatory ratio new;
label geography ='unit of geography';
label confirmatory ='Total dx past 4 weeks';
label ratio = "Signal Strength (# of SDs above mean)";
label new='* indicates new signal since last week';
title1 "Trends Report based on diagnosis date";
title2      ;
title3 'The trends report compares the count of all cases (except contacts
and possible exposures)';
title4 'diagnosed in the past 4 weeks to the mean and standard deviation of
cases diagnosed';
title5 'during similar time periods in the past 5 years.';
title6 ;
title7 "This report was created
%sysfunc(left(%qsysfunc(date(),worddate18.)))";
footnote1 font='Arial' height=1 "Total dx past 4 weeks includes only
confirmed, probable, suspect and pending case statuses";
footnote2 font='Arial' height=1 "When # of SD's above mean > 2, current
period is considered a signal";
```

```
footnote3 font='Arial' height=1 "A missing signal strength value indicates an
SD = 0 in the baseline period";
run;


*************************************************************;
* The following code preps data for graphs of signals
*************************************************************;

***** CITYWIDE *****;
* fill in missing Saturdays with zeroes for all diseases;
proc transpose data = current_data(rename=(events_adj=events)) out = trans;
      by disease_code;
      id fsatdiag;
      var events;
      format fsatdiag 8.;
run;
proc transpose data = trans out = trans2;
      by disease_code;
run;
data collapsed_events_fillin;
      set trans2;
      if events = . then events = 0;
      fsatdiag = input(substr(_name_,2),8.);
      format fsatdiag mmddyy10.;
      drop _name_;
      if fsatdiag < date();
run;
proc sort data = collapsed_events_fillin; by disease_code fsatdiag; run;
* create a moving sum of events for the previous four weeks;
data events_4wk_moving_sum;
      set collapsed_events_fillin;
      by disease_code;
      retain num_sum 0;
      if first.disease_code then do;
      count=0;
            num_sum=0;
      end;
      count+1;
      last4=lag4(events);
      if count gt 4 then num_sum=sum(num_sum,events,-last4);
      else num_sum=sum(num_sum,events);
      drop count last4;
run;
* append to adjusted 4-wk sum of event counts;
data events_4wk_moving_sum;
set events_4wk_moving_sum(where=(fsatdiag>&lastday_BL) drop=events)
      signals.adjusted_baseline_city(rename=(num_sum_adj=num_sum)
      keep=disease_code fsatdiag num_sum_adj);
run;
proc sort data = events_4wk_moving_sum; by disease_code fsatdiag; run;
* pull signals for this week;
proc sort data = AllSignificant out = trends_signals;
      by disease_code fsatdiag;
      where geography = "City";
run;
```

```
* merge signal data with event counts;
data events_and_signals;
      merge trends_signals events_4wk_moving_sum;
      by disease_code fsatdiag;
run;
proc sort data = events_and_signals;
      by disease_code fsatdiag rundate;
run;
* create a record for the current mean and low and high interval that the
current count is being compared to;
data reshape;
      set events_and_signals;
      where fsatdiag = &maxfsatdiag & mean ~= .;
      yvar = mean; num_sum = current; output;
      yvar = mean - sd; num_sum = .d; output;
      yvar = mean + sd; num_sum = .d; output;
run;
* output the rest of the dataset (minus the current information);
data therest;
      set events_and_signals;
      where (fsatdiag ~= &maxfsatdiag | mean = .) & fsatdiag >= (date()-
            365*5-60);
      yvar = .;
      mean = .;
      current = .;
run;
* append the current data to the rest of the data;
proc append base = reshape data = therest;
run;
data reshape_city;
      set reshape;
      if &maxfsatDiag-365-7  <= fsatdiag <= &maxfsatDiag-365 then
            period1=fsatdiag;
      if &maxfsatDiag-730-7  <= fsatdiag <= &maxfsatDiag-730 then
      period2=fsatdiag;
      if &maxfsatDiag-365*3-7  <= fsatdiag <= &maxfsatDiag-365*3 then
            period3=fsatdiag;
      if &maxfsatDiag-365*4-7  <= fsatdiag <= &maxfsatDiag-365*4 then
            period4=fsatdiag;
      if &maxfsatDiag-365*5-7  <= fsatdiag <= &maxfsatDiag-365*5 then
            period5=fsatdiag;
      geounit = "City";
      geog = "NYC";
run;
* sort for graphing;
proc sort data = reshape_city(keep=disease_code geounit geog fsatdiag num_sum
      current yvar mean period:);
      by disease_code fsatdiag;
run;

** The equivalent analysis above is carried out at all geographic resolutions
(in our case at Borough and UHF neighborhood);

* append all geographic levels;
data reshape_all;
      length geog $50.;
```

```
      set reshape_city reshape_boro reshape_uhf;
run;


************************************************************;
* The following code preps data for table 2 in the linelist reports;
************************************************************;

***** CITYWIDE *****;
* pull in raw data going back 5 years;
proc sql;
      create table raw_data as
      select disease_code, disease, fsatdiag, count(event_id) as events
      from event_level_input
      where disease_status in ("CONFIRMED","PROBABLE","SUSPECT")
      group by disease_code, disease, fsatdiag
      order by disease_code, disease, fsatdiag;
quit;
* reshape to fill in all dates for all diseases;
proc transpose data = raw_data out = trans;
      by disease_code disease;
      id fsatdiag;
      var events;
      format fsatdiag 8.;
run;
proc transpose data = trans out = trans2;
      by disease_code disease;
run;
data raw_data_fillin;
      set trans2;
      if events = . then events = 0;
      fsatdiag = input(substr(_name_,2),8.);
      format fsatdiag mmddyy10.;
      drop _name_;
run;
proc sort data = raw_data_fillin; by disease_code disease fsatdiag; run;
* save current week's dates for labels in output document;
proc sql noprint;
      select put(max(fsatdiag),date8.), put(max(fsatdiag)-27,date8.)
      into :weekmax, :weekmin
      from raw_data_fillin
quit;
* merge current data with baseline data and categorize weeks into relevant
time periods for analysis;
data raw_city;
      set raw_data;
      length week $50.;
      if events = . then events = 0;
      year = cat("year",put(year(fsatdiag),$4.));
      if (fsatdiag >= (date()-27) & fsatdiag <= (date()-21)) |
            (fsatdiag >= (date()-365-27) & fsatdiag <= (date()-365-21)) |
            (fsatdiag >= (date()-365*2-27) & fsatdiag <= (date()-365*2-21)) |
            (fsatdiag >= (date()-365*3-27) & fsatdiag <= (date()-365*3-21)) |
            (fsatdiag >= (date()-365*4-27) & fsatdiag <= (date()-365*4-21)) |
            (fsatdiag >= (date()-365*5-27) & fsatdiag <= (date()-365*5-21))
            then week='3 weeks ago';
```

```
      if (fsatdiag >= (date()-20) & fsatdiag <= (date()-14)) |
            (fsatdiag >= (date()-365-20) & fsatdiag <= (date()-365-14)) |
            (fsatdiag >= (date()-365*2-20) & fsatdiag <= (date()-365*2-14)) |
            (fsatdiag >= (date()-365*3-20) & fsatdiag <= (date()-365*3-14)) |
            (fsatdiag >= (date()-365*4-20) & fsatdiag <= (date()-365*4-14)) |
            (fsatdiag >= (date()-365*5-20) & fsatdiag <= (date()-365*5-14))
            then week='2 weeks ago';
      if (fsatdiag >= (date()-13) & fsatdiag <= (date()-7)) |
            (fsatdiag >= (date()-365-13) & fsatdiag <= (date()-365-7)) |
            (fsatdiag >= (date()-365*2-13) & fsatdiag <= (date()-365*2-7)) |
            (fsatdiag >= (date()-365*3-13) & fsatdiag <= (date()-365*3-7)) |
            (fsatdiag >= (date()-365*4-13) & fsatdiag <= (date()-365*4-7)) |
            (fsatdiag >= (date()-365*5-13) & fsatdiag <= (date()-365*5-7))
            then week='1 week ago';
      if (fsatdiag >= (date()-6) & fsatdiag <= (date())) |
            (fsatdiag >= (date()-365-6) & fsatdiag <= (date()-365)) |
            (fsatdiag >= (date()-365*2-6) & fsatdiag <= (date()-365*2)) |
            (fsatdiag >= (date()-365*3-6) & fsatdiag <= (date()-365*3)) |
            (fsatdiag >= (date()-365*4-6) & fsatdiag <= (date()-365*4)) |
            (fsatdiag >= (date()-365*5-6) & fsatdiag <= (date()-365*5))
            then week='Most recent week';
      if week ~= "";
run;
proc sort data = raw_city; by disease_code disease week year; run;
proc transpose data = raw_city out = trans;
      by disease_code disease week;
      var events;
      id year;
run;
proc transpose data = trans out = trans2 (rename=(_name_=year1));
      by disease_code disease week;
run;
proc sort data = trans2; by disease_code disease year1; run;
proc transpose data = trans2 out = final_city (drop=_name_);
      by disease_code disease year1;
      var events;
      id week;
run;
data final_city2;
      retain disease_code disease geog year _3_weeks_ago _2_weeks_ago
            _1_week_ago Most_recent_week;
      set final_city;
      array weeks _3_weeks_ago _2_weeks_ago _1_week_ago Most_recent_week;
      do over weeks;
            if weeks = . then weeks = 0;
      end;
      length year $30.;
      year = cat(substr(year1,5)," Conf/Prob/Susp");
      geog = "NYC";
      label _3_weeks_ago = "4 weeks ago";
      label _2_weeks_ago = "3 weeks ago";
      label _1_week_ago = "2 weeks ago";
      label Most_recent_week = "Most recent week (Sun-Sat)";
      drop year1;
run;
proc sql;
```

```
      create table alldiseases as select distinct disease_code, disease,
            max(substr(year,1,4)) as year2
      from final_city2 group by disease_code order by disease_code;
quit;

* count pending separately;
proc sql;
      create table raw_pending as
      select disease_code, disease, fsatdiag, count(event_id) as pending
      from event_level_input
      where disease_status = "PENDING" & year(fsatdiag) = year(date())
      group by disease_code, disease, fsatdiag
      order by disease_code, disease, fsatdiag;
quit;
* reshape to fill in all dates for all diseases;
proc transpose data = raw_pending out = trans;
      by disease_code disease;
      id fsatdiag;
      var pending;
      format fsatdiag 8.;
run;
proc transpose data = trans out = trans2;
      by disease_code disease;
run;
data raw_pending_fillin;
      set trans2;
      if pending = . then pending = 0;
      fsatdiag = input(substr(_name_,2),8.);
      format fsatdiag mmddyy10.;
      drop _name_;
run;
proc sort data = raw_pending_fillin; by disease_code disease fsatdiag; run;
* merge current data with baseline data and categorize weeks into relevant
time periods for analysis;
data raw_city_pending;
      set raw_pending_fillin;
      length week $50.;
      if pending = . then pending = 0;
      year = cat("year",put(year(fsatdiag),$4.));
      if (fsatdiag >= (date()-27) & fsatdiag <= (date()-21)) |
            (fsatdiag >= (date()-365-27) & fsatdiag <= (date()-365-21)) |
            (fsatdiag >= (date()-365*2-27) & fsatdiag <= (date()-365*2-21)) |
            (fsatdiag >= (date()-365*3-27) & fsatdiag <= (date()-365*3-21)) |
            (fsatdiag >= (date()-365*4-27) & fsatdiag <= (date()-365*4-21)) |
            (fsatdiag >= (date()-365*5-27) & fsatdiag <= (date()-365*5-21))
            then week='3 weeks ago';
      if (fsatdiag >= (date()-20) & fsatdiag <= (date()-14)) |
            (fsatdiag >= (date()-365-20) & fsatdiag <= (date()-365-14)) |
            (fsatdiag >= (date()-365*2-20) & fsatdiag <= (date()-365*2-14)) |
            (fsatdiag >= (date()-365*3-20) & fsatdiag <= (date()-365*3-14)) |
            (fsatdiag >= (date()-365*4-20) & fsatdiag <= (date()-365*4-14)) |
            (fsatdiag >= (date()-365*5-20) & fsatdiag <= (date()-365*5-14))
            then week='2 weeks ago';
      if (fsatdiag >= (date()-13) & fsatdiag <= (date()-7)) |
            (fsatdiag >= (date()-365-13) & fsatdiag <= (date()-365-7)) |
            (fsatdiag >= (date()-365*2-13) & fsatdiag <= (date()-365*2-7)) |
```

```
            (fsatdiag >= (date()-365*3-13) & fsatdiag <= (date()-365*3-7)) |
            (fsatdiag >= (date()-365*4-13) & fsatdiag <= (date()-365*4-7)) |
            (fsatdiag >= (date()-365*5-13) & fsatdiag <= (date()-365*5-7))
            then week='1 week ago';
      if (fsatdiag >= (date()-6) & fsatdiag <= (date())) |
            (fsatdiag >= (date()-365-6) & fsatdiag <= (date()-365)) |
            (fsatdiag >= (date()-365*2-6) & fsatdiag <= (date()-365*2)) |
            (fsatdiag >= (date()-365*3-6) & fsatdiag <= (date()-365*3)) |
            (fsatdiag >= (date()-365*4-6) & fsatdiag <= (date()-365*4)) |
            (fsatdiag >= (date()-365*5-6) & fsatdiag <= (date()-365*5))
            then week='Most recent week';
      if week ~= "";run;
proc sort data = raw_city_pending; by disease_code disease week year; run;
proc transpose data = raw_city_pending out = trans;
      by disease_code disease week;
      var pending;
      id year;
run;
proc transpose data = trans out = trans2 (rename=(_name_=year1));
      by disease_code disease week;
run;
proc sort data = trans2; by disease_code disease year1; run;
proc transpose data = trans2 out = final_city_pending (drop=_name_);
      by disease_code disease year1;
      var pending;
      id week;
run;
data final_city_pending2;
      retain disease_code disease geog year _3_weeks_ago _2_weeks_ago
            _1_week_ago Most_recent_week;
      merge final_city_pending alldiseases;
      by disease_code disease;
      array weeks _3_weeks_ago _2_weeks_ago _1_week_ago Most_recent_week;
      do over weeks;
            if weeks = . then weeks = 0;
      end;
      length year $30.;
      if year1 ~= "" then year = cat(substr(year1,5)," Pending");
      else year = cat(strip(year2)," Pending");
      geog = "NYC";
      drop year1 year2;
      if ~(_3_weeks_ago=0 & _2_weeks_ago=0 & _1_week_ago=0 &
            Most_recent_week=0);
run;
data final_city;
      set final_city2 final_city_pending2;
run;
proc sort data = final_city; by disease_code disease geog descending year;
run;

** The equivalent code above is carried out at all geographic resolutions (in
our case at Borough and UHF neighborhood);

data final_freqs;
      retain disease_code disease geog year _3_weeks_ago _2_weeks_ago
            _1_week_ago Most_recent_week;
```

```
      length geog $50.;
      set final_city final_boro final_uhf;
      Total = _3_weeks_ago + _2_weeks_ago + _1_week_ago + Most_recent_week;
run;


*************************************************************;
* This code creates output reports with signal details;
*************************************************************;

* create folders for output to be saved;
options noxwait;
x "cd ...\&fileweek\";
x "md Linelist";
x "cd ...\&fileweek\Linelist";
x "md Lab_Results";

*-- signals by city, boro and uhf;
%macro rollup(level=,merge=);
proc sql noprint;
      create table signals_&level as
      select *
      from allsignificant
      where geounit="&level";
quit;

*-- linelist by city, boro and uhf;
* while the analysis is based on all disease statuses, only confirmatory
cases are printed in the linelist;
proc sort data = signals_&level (keep=&merge geography metric confirmatory
      mean sd ratio rate); by &merge; run;
proc sort data = event_level_input out = event_level_input2
      (keep=event_id disease_code disease patinit disease_status
            investigation_status diagnosis_date gender
            age_years street_1 boro zip uhfname x_coord y_coord);
      by &merge;
      where fsatdiag >= (&maxfsatdiag-22) & fsatdiag <= &maxfsatdiag &
            disease_status in("CONFIRMED","PROBABLE","SUSPECT","PENDING");
run;
data linelist_&level;
      merge signals_&level (in=insignals) event_level_input2;
      by &merge;
      if insignals;
      level = "&level";
run;
%mend rollup;
%rollup(level=City,merge=disease_code);
%rollup(level=Boro,merge=disease_code boro);
%rollup(level=UHF,merge=disease_code uhfname);
* save events included in signals to permanent file for future references;
data signals.linelist_&fileweek;
      set linelist_city linelist_boro linelist_uhf;
      keep disease_code event_id geography;
run;

* annotation for map;
```

```
data signals;
      length function style color $ 8 text $ 20 geocode $ 3;
      retain xsys ysys '2' hsys '3' when 'a';
      set linelist_boro linelist_city linelist_uhf;
      x = input(x_coord,12.);
      y = input(y_coord,12.);
      function='label'; style='arial'; text='+'; size=1.5; color = "red";
      if level = "UHF" then geog = uhfname;
      if level = "Boro" then geog = boro;
      if level = "City" then geog = "NYC";
      if x = . & y = . then geocode = "no";
      else geocode = "yes";
run;
proc sort data = signals; by disease_code geography diagnosis_date event_id;
run;
* add a count variable to number each case within each signal;
data signals;
      set signals;
      count + 1;
      by disease_code geography;
      if first.disease_code | first.geography then count = 1;
      text = strip(put(count,$3.));
run;
* merge this week's linelist with last week's linelist to identify new cases
in signals that existed in the prior week;
proc sort data = signals.linelist_&lastweek out = lastweek; by disease_code
      geography event_id; run;
proc sort data = signals; by disease_code geography event_id; run;
data signals;
      merge lastweek (in=a) signals (in=b);
      by disease_code geography event_id;
      if b;
      if b & ~a then new = "*";
      if b & ~a then newnum = 1;
      else newnum = 0;
run;
proc sort data = signals; by disease_code geography diagnosis_date; run;

* create macro variables to facilitate looping through signals below;
proc sort data = allsignificant_compare (keep=disease_code disease geounit
boro uhfname new) out = mapsignificant; by disease_code disease geounit boro
uhfname; run;
data _null_;
      set mapsignificant;
      length geog $42.;
      by disease_code disease geounit boro uhfname;
      geog = boro;
      if geog = "" then geog = uhfname;
      if geounit="City" then geog = "NYC";
      where ~(geounit="Boro" & boro in("UNKNOWN" ""));
      if first.disease | first.geounit | first.boro | first.uhfname then do;
            i+1;
            ii=left(put(i,20.));
            call symputx ('disease'||ii,strip(disease));
            call symputx ('diseasecode'||ii,strip(disease_code));
            call symputx ('geounit'||ii,strip(geounit));
```

```
            call symputx ('geog'||ii,strip(geog));
            call symputx ('geog2'||ii,substr(strip(geog),1,3));
            call symputx ('total',put(ii,20.));
            call symputx ('new'||ii,strip(new));
      end;
run;

*-- create summary dataset for choropleth map;
* read in shapefile;
proc mapimport datafile=…/Maps/zip_code_areas_w_uhf.shp' out = uhfmap;
run;
data uhfpop;
      set /* read in a dataset with each geographic unit in map and the
            corresponding population */;
run;
* calculate the number of events in the last four weeks without missing zip
code by disease and geographic unit;
proc sql;
      create table disease_summ as
      select disease_code, disease, input(uhfcode,3.) as uhfcode, count(*) as
            reports
      from event_level_input2
      where (disease_status in("PENDING","CONFIRMED","PROBABLE","SUSPECT") &
            year(fsatdiag) = year(date()) & week(fsatdiag) < week(date()) &
            week(fsatdiag) >= week(date()) - 4 & zip ~= "")
      group by uhfcode, disease_code, disease
      order by uhfcode, disease_code, disease;
quit;
* calculate rates by geographic unit for mapping;
data disease_summ_rates;
      merge disease_summ(in=indisease) uhfpop(in=inuhfpop);
      by uhfcode;
      if indisease & inuhfpop & census_pop ~= 0;
      rate = round((reports / census_pop) * 100000,.01);
run;

* loop through signals to create a report for each one;
%macro linelist;
%do i=1 %to &total;
* subset datasets to the relevant information for each signal;
data &&diseasecode&i;
   set disease_summ_rates;
   where disease = "&&disease&i";
run;
data signals&i;
   set signals;
   where disease = "&&disease&i" & level = "&&geounit&i" & geog =
      "&&geog&i";
run;
data final_freqs_&i;
   set final_freqs;
   where disease = "&&disease&i" & geog = "&&geog&i";
run;
* summary data for first page;
proc sql;
            create table signals2_&i as
```

```
      select distinct disease, geography, metric, confirmatory, mean,
            sd,ratio, rate, cityrate, count(new) as new_count,
            min(newnum) as new_signal
      from signals&i
      group by disease, geography, metric;
quit;
proc format; value na .="N/A"; run;
data signals2_&i;
      length new_signal2 $3.;
      set signals2_&i;
      if new_signal = 1 then new_signal2 = "yes";
      else new_signal2 = "no";
      if new_signal2 = "yes" then new_count = .;
      format new_count na.;
run;

ods noresults;
options orientation=landscape;
* output results to Linelist folder;
ods rtf
file="...\&fileweek\Linelist\weeklylinelist_&&disease&i.._&&geog&i...rt
f" bodytitle;

* PAGE 1 - summary of signal;
title "&&disease&i";
title2 "&&geounit&i Signal in &&geog&i";
footnote1 font='Arial' height=1 "Total dx past 4 weeks includes only
      confirmed, probable, suspect and pending case statuses";
footnote2 font='Arial' height=1 "When # of SDs above mean > 2, current
      period is considered a signal";
footnote3 font='Arial' height=1 "A missing signal strength value
      indicates an SD = 0 in the baseline period";
ods proclabel="&&geounit&i Signal in &&geog&i";
proc report data=signals2_&i nowd style(report)={outputwidth=7in
      font_size=10pt};
      columns disease geography metric confirmatory ratio new_signal2
            new_count rate cityrate;
      define disease/display "Disease" width=20;
      define geography/display "Unit of geography" width=20;
      define metric/display "Date of interest" width=20;
      define confirmatory/display "Total dx past 4 weeks: &weekmin -
            &weekmax" width=20;
      define ratio/display "Signal Strength (# of SDs above mean)"
            width=10;
      define new_signal2/display "new signal since last week?"
            width=10;
      define new_count/display "if not new, how many new events in
            signal?" width=15;
run;

* PAGE 2 - raw counts;
footnote1 font='Arial' height=1 "Unadjusted counts of cases by year,
      week and case status";
footnote2;
proc report data=final_freqs_&i nowd style(report)={outputwidth=7in};
```

```
      columns year Most_recent_week _1_week_ago _2_weeks_ago
      _3_weeks_ago Total;
run;

* PAGE 3 - map;
* subset signals dataset to those that are not missing x and y
      coordinates for mapping;
data nomiss_signals&i;
      set signals&i;
      where x ~= . & y ~= .;
run;
footnote1;
pattern1 v=s c=grayff;
pattern2 v=s c=graydd;
pattern3 v=s c=graybb;
pattern4 v=s c=gray88;
pattern5 v=s c=gray66;
goptions reset=goptions device=png300 target=png300 ftext='Arial'
      htext=1 ftitle='Arial/bold' htitle=1.5 xmax=9 in ymax=7 in;
legend1 label= (j=l font='Arial/bold' 'Rate per 100,000 for previous 4
      wks'
  j=l font='Arial' '*Numbers indicate the location of events in the
      signal.'
  j=l '*Note that rates are meant to provide context only and do'
  j=l '      not necessarily correspond to signals.'
  position=(top left)) across=1 down=5 frame position=(bottom outside);
proc gmap data = &&diseasecode&i map = uhfmap anno=nomiss_signals&i;
  id uhfcode;
  choro rate / levels=5 coutline=black legend=legend1 cdefault=white;
run;
quit;

* PAGE 4 - line list;
ods proclabel="Line List";
* if the signal is not new, include an extra column to indicate which
 events are newly added to the repeated signal;
%if "&&new&i" = "" %then %do;
proc report data=signals&i nowd;
      columns new text event_id patinit disease_status
            investigation_status diagnosis_date gender age_years
            street_1 boro zip uhfname geocode;
      define new/ display "New" width=1;
      define text/ display "#" width=2;
      define event_id/ display "Event ID" width=10;
      define patinit/display "Pat. Init." width=2;
      define disease_status/display "Disease Status" width=4;
      define investigation_status/display "Investigation Status"
            width=4;
      define diagnosis_date/display "Diagnosis Date" width=10;
      define gender/display "Gender" width=1;
      define age_years/display "Age" width=3;
      define street_1/display "Address" width=10;
      define boro/display "Boro" width=12;
      define zip/display "Zip" width=5;
      define uhfname/display "UHF" width=10;
      define geocode/display "Geocode" width=3;
```

```
    run;
    %end;
    %if "&&new&i" = "*" %then %do;
    proc report data=signals&i nowd;
          columns text event_id patinit disease_status investigation_status
                diagnosis_date gender age_years street_1 boro zip uhfname
                geocode;
          define text/ display "#" width=2;
          define event_id/ display "Event ID" width=10;
          define patinit/display "Pat. Init." width=2;
          define disease_status/display "Disease Status" width=4;
          define investigation_status/display "Investigation Status"
                width=4;
          define diagnosis_date/display "Diagnosis Date" width=10;
          define gender/display "Gender" width=1;
          define age_years/display "Age" width=3;
          define street_1/display "Address" width=10;
          define boro/display "Boro" width=12;
          define zip/display "Zip" width=5;
          define uhfname/display "UHF" width=15;
          define geocode/display "Geocode" width=3;
    run;
    %end;

    * PAGE 6 - graph;
    proc sql;
          create table graphit as
          select *
          from reshape_all
          where disease_code="&&diseasecode&i" and geounit="&&geounit&i"
                and geog="&&geog&i";
* allow axes to be flexible depending on counts;
    proc sql noprint;
          select max(num_sum), min(min(yvar,num_sum)), max(fsatdiag)
                format=mmddyy10., max(period1), max(period2), max(period3),
                max(period4), max(period5)
          into :maxsignal, :minsignal, :maxweek, :period1, :period2,
                :period3, :period4, :period5
          from graphit;
    quit;
    data _null_;
            if &maxsignal.<=10 then do; maxaxis=10; intaxis=1; end;
            else if 10<&maxsignal.<=25 then do; maxaxis=25; intaxis=5; end;
            else if 25<&maxsignal.<=50 then do; maxaxis=50; intaxis=5; end;
            else if 50<&maxsignal.<=100 then do; maxaxis=100; intaxis=10;
                end;
            else if 100<&maxsignal.<=500 then do; maxaxis=500; intaxis=50;
                end;
            else if 500<&maxsignal.<=1000 then do; maxaxis=1000;
                intaxis=100; end;
            else if 1000<&maxsignal.<=1500 then do; maxaxis=1500;
                intaxis=150; end;
            else if 1500<&maxsignal.<=2000 then do; maxaxis=2000;
                intaxis=200; end;
            else if 2000<&maxsignal.<=2500 then do; maxaxis=2500;
                intaxis=250; end;
```

```
        else if 2500<&maxsignal.<=3000 then do; maxaxis=3000;
            intaxis=300; end;
        else if 3000<&maxsignal.<=3500 then do; maxaxis=3500;
            intaxis=350; end;
        else if 3500<&maxsignal.<=4000 then do; maxaxis=4000;
            intaxis=400; end;
        else if 4000<&maxsignal.<=4500 then do; maxaxis=4500;
            intaxis=450; end;
        else if 4500<&maxsignal.<=5000 then do; maxaxis=5000;
            intaxis=500; end;
        if &minsignal.<0 & &minsignal>-1 then do; minaxis=-1; end;
        else if &minsignal.<-1 & &minsignal>-5 then do; minaxis=-5;
            end;
        else minaxis = 0;
%global maxaxis intaxis;
  call symput('minaxis',minaxis);
  call symput('maxaxis',maxaxis);
  call symput('intaxis',intaxis);
run;
symbol1 i=hiloctj color=blue line=2;
symbol2 i=none color=blue value=dot height=1.5;
symbol3 i=l color=black value=none height=1.5;
symbol4 i=none color=red value=dot height=1.5;
legend1 label=none order=("mean" "current" "num_sum")
      value=(h=2 pct f=simplex j=c c=black 'Mean +/- 2 SD' 'Signal'
      'Moving 4-Week Sum');
axis1 label=none color=black
    value=(h=2 pct f=simplex j=c c=black)
      order=(&minaxis. to &maxaxis. by &intaxis.)
      reflabel=(color=black)
      width=1
      length=75 pct
      major=none minor=none;
axis2 label=none color=black
    value=(h=2 pct f=simplex j=c c=black)
      reflabel=(color=black)
      width=1
      major=(number=6) minor=(number=12);
goptions reset=goptions device=png300 target=png300 ftext='Arial'
      htext=1 ftitle='Arial/bold' htitle=1.5 xmax=10 in ymax=6.5 in;
proc gplot data=graphit;
      title "&&disease&i";
      title2 "&&geounit&i Signal in &&geog&i";
      title3 height=1.05 "Moving 4-wk sum of adjusted case counts
            ending &maxweek";
      plot (yvar mean num_sum current)*fsatdiag / cframe=GWH autovref
            cvref=wh overlay skipmiss vaxis=axis1 haxis=axis2
            legend=legend1 lhref=2 chref=stro href=&period1 &period2
            &period3 &period4 &period5;
      footnote1 height=0.75 "Disease status includes all except Contact
            and Possible Exposure";
      footnote3 height=0.75 "Vertical dotted lines represent
            corresponding 4-wk periods in baseline";
run;
quit;
ods rtf close;
```

```
%end;
%mend;
%linelist;


************************************************************;
* The following code emails signal information to reviewers.
************************************************************;

* read in list of email recipients by disease;
proc import datafile = "…\Data\reviewers.xls" out = reviewers replace dbms =
      excel; run;
* merge with signal information;
proc sql noprint;
create table signals_reviewer as
select distinct s.disease_code
      ,s.disease
      ,propcase(s.geog) as geography
      ,count(*) as cases_in_signal
      ,case (min(s.newnum)) when 0 then 'No' else 'Yes' end as new_signal
      ,case (min(s.newnum)) when 0 then count(s.new) else . end as new_cases
      ,p.notes as REVIEWER label=''
from signals as s left join reviewers as p on s.disease_code=p.code
where s.suppress = 'no'
group by s.disease_code, s.geography
order by p.notes;
quit;

* create macro to send emails;
%macro email;
data _null_;
set signals_reviewer;
by reviewer;
if first.reviewer then do;
            i+1;
            call symputx('reviewer'||left(put(i,2.)),reviewer);
            call symputx('end',left(put(i,2.)));
end;
run;
* loop through reviewers to send all signal information in one email;
%do i=1 %to &end;
      data reviewer&i;
            set signals_reviewer;
            where reviewer = "&&reviewer&i";
      run;
* &name macro set at beginning of code by whoever is running it;
FILENAME outbox EMAIL
                from= "&name@health.nyc.gov"
                        to=(&&reviewer&i)
                        cc=("&name@health.nyc.gov")
                        subject="Maven: AOW Signals for &fileweek"
                type='text/html'
                CT ='text/html';
ods html body=outbox style=minimal;
ods escapechar='^';
```

```
ods text = "^{style [just=l]AOW Signals for &fileweek}";
* print summary table of signals in body of email;
proc report data=reviewer&i nowd nocenter spacing=5;
      columns disease geography cases_in_signal new_signal new_cases;
      define disease/display "Disease" ;
      define geography/display "Geography" ;
      define cases_in_signal/display "total dx past 4 weeks" ;
      define new_signal/display "new signal since last week?" ;
      define new_cases/display "if not new, how many new events in signal?" ;
      title;
      footnote;
run;
* print link to line lists in body of email;
ods text = "^{style [just=l]Signal details and the linelists are here: }";
ods html text = "^{style [just=l] …\&fileweek\Linelist\ }";
ods text = "^{style [just=l] If you have questions, notify the analyst.}";
ods html close;
%end;
%mend;

%email;
```
